# Feasibility study of a novel general purpose CZT-based digital SPECT camera: initial clinical results

**DOI:** 10.1186/s40658-018-0205-z

**Published:** 2018-03-14

**Authors:** Elinor Goshen, Leonid Beilin, Eli Stern, Tal Kenig, Ronen Goldkorn, Simona Ben-Haim

**Affiliations:** 1 Department of Nuclear Medicine, Chaim Sheba Medical Center, Tel Hashomer, Ramat Gan, Israel; 2 Sackler School of Medicine, Tel Aviv University, Tel Aviv, Israel; 3 Molecular Dynamics, Hamilton, Bermuda; 4 Department of Nuclear Cardiology, Chaim Sheba Medical Center, Tel Hashomer, Ramat Gan, Israel; 5 Institute of Nuclear Medicine, University College London and UCL Hospitals, London, UK

**Keywords:** CZT, General purpose, SPECT, Clinical

## Abstract

**Background:**

The performance of a prototype novel digital single-photon emission computed tomography (SPECT) camera with multiple pixelated CZT detectors and high sensitivity collimators (Digital SPECT; Valiance X12 prototype, Molecular Dynamics) was evaluated in various clinical settings.

Images obtained in the prototype system were compared to images from an analog camera fitted with high-resolution collimators. Clinical feasibility, image quality, and diagnostic performance of the prototype were evaluated in 36 SPECT studies in 35 patients including bone (*n* = 21), brain (*n* = 5), lung perfusion (*n* = 3), and parathyroid (*n* = 3) and one study each of sentinel node and labeled white blood cells. Images were graded on a scale of 1–4 for sharpness, contrast, overall quality, and diagnostic confidence.

**Results:**

Digital CZT SPECT provided a statistically significant improvement in sharpness and contrast in clinical cases (mean score of 3.79 ± 0.61 vs. 3.26 ± 0.50 and 3.92 ± 0.29 vs. 3.34 ± 0.47 respectively, *p* < 0.001 for both). Overall image quality was slightly higher for the digital SPECT but not statistically significant (3.74 vs. 3.66).

**Conclusion:**

CZT SPECT provided significantly improved image sharpness and contrast compared to the analog system in the clinical settings evaluated. Further studies will evaluate the diagnostic performance of the system in large patient cohorts in additional clinical settings.

## Background

Gamma cameras with digital solid-state cadmium zinc telluride (CZT) detectors and tungsten collimators have been reported to provide improved images when compared to those produced by conventional analog cameras, fitted with thallium-doped sodium iodide (NaI [Tl]) detectors and lead collimators [1, 2]. In particular, dedicated cardiac single-photon emission computed tomography (SPECT) cameras with CZT detectors have been commercially available for over a decade, and the published data regarding their potential superiority over conventional gamma cameras is rapidly accumulating [1–10].

The advantage of CZT SPECT technology is due to a number of contributing factors and cannot be attributed simply to the intrinsic detection efficiency of the CZT material, which is actually quite similar to that of NaI [4]. The intrinsic spatial resolution of CZT systems is better than NaI systems, firstly due to the pixelated nature of the detectors. The digital CZT SPECT technology has improved energy resolution compared to scintillation-based systems, which can facilitate better scatter correction and simultaneous multiple isotope scanning protocols. In addition, there is a significant improvement of energy resolution using CZT detectors, 5% at 140 keV compared to 10% with a conventional camera [5–7]. Another intrinsic advantage of digital CZT detection technology over analog technology is the significantly higher count rate afforded by the new technology [3], which facilitates dynamic SPECT [8, 9], together with significant reductions in radiation exposure and imaging times [10]. In addition, the light weight and small footprint of the units enable non-standard detector configurations and system architectures to attain performance improvements. In particular, the small form of CZT detectors allows for improved proximity to the patient and richer angular sampling by facilitating complex mechanical manipulation of the detectors. The improved proximity and sampling coupled with proprietary reconstruction algorithms results in further improvement to spatial resolution when compared to conventional SPECT systems, even when the digital system is equipped with high-sensitivity collimators [1, 3].

While demonstrated to enable better spatial resolution, shorter acquisition times, and/or reduced administered doses, the use of CZT-based digital cameras have so far been limited to cardiac imaging, and only sparse data exist regarding other nuclear medicine applications [11]. In this feasibility study, 35 patients injected with various technetium-labeled pharmaceuticals were imaged in a prototype general purpose digital SPECT scanner (Valiance X12 prototype, Molecular Dynamics) for non-cardiac clinical indications.

The novel CZT detector-based general purpose scanner utilizes detectors similar to those incorporated in the well-established D-SPECT camera [1] but placed on a ring-shaped gantry. In addition, the hardware provides for unique scanning geometry, particularly stemming from the independent radial and swivel motion of the detectors. The system is also equipped with proprietary reconstruction software, implementing advanced algorithms and system modeling.

In order to evaluate the performance of this novel CZT system fitted with multiple pixelated CZT detectors, clinical images were obtained in the digital SPECT camera and compared to those obtained in an analog system routinely used in our department.

## Methods

### Cameras and study design

The study was designed to validate the clinical feasibility of the novel digital SPECT system (Valiance X12 prototype) and compare the overall clinical performance of the digital system to that of analog systems routinely used.

The analog cameras used for comparison were the Discovery NM/CT 670 and Infinia Hawkeye 4 (GE Healthcare) fitted with low-energy high-resolution (LEHR) collimators. Images were reconstructed using the standard protocols used in our department, with the vendor-provided ordered subsets expectation minimization (OSEM) iterative reconstruction (all except brain studies) or filtered back-projection (FBP) reconstruction (brain studies).

**Fig. 1:**
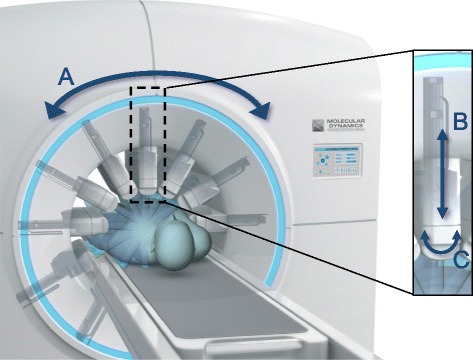
Flexible architecture of the Valiance X12 enables multiple independent axes of motion. (A) Gantry rotation, (B) radial detector motion, and (C) swivel motion. The complex motion facilitates multiple angular acquisition capabilities, with numerous different viewing angles covering the target

The digital Valiance X12 prototype (Fig. 1) is comprised of CZT-based elongated detectors, 16 by 64 pixels each, covering approximately 4 by 16cm, mounted on a ring-shaped gantry. The detectors are electronically pixelated and are equipped with high-sensitivity tungsten parallel-hole collimators, with a wider opening solid angle than the analog SPECT LEHR collimator (about two times wider). The square collimator holes are in registration with the pixel array. The system’s architecture provides radial and swivel motion of the detector, which allows for targeted region of interest (ROI) centered imaging. Images are produced using a proprietary iterative reconstruction engine, based on an ordered subsets maximum a-posteriori (OS-MAP) algorithm, which accurately models the system and acquisition geometry.

Thirty-five patients (21 male, 14 female, mean age 50 ± 17 years, range 19–74 years) were scanned. All patients underwent planar and SPECT (or SPECT/CT) imaging in the analog SPECT camera, immediately followed by SPECT in the Valiance X12 prototype. Overall, a total of 36 analog SPECT scans were available (30 Infinia Hawkeye 4, 6 Discovery NM/CT 670).

Twenty patients underwent ^99m^Tc MDP SPECT scans of 21 regions on both analog and digital SPECT. Skeletal regions evaluated included the knees (*n* = 8), lumbar spine, pelvis and hips (*n* = 5), ankles and feet (*n* = 5), skull (*n* = 1), cervical spine (*n* = 1), and tibia (*n* = 1). One patient had two skeletal regions evaluated. The same patient also underwent both standard and targeted ROI-centric scans on the digital SPECT camera in the same session resulting in 23 bone scans acquired by digital SPECT and 21 by analog SPECT.

Additional SPECT studies included ^99m^Tc-ECD brain perfusion (*n* = 5), ^99m^Tc-MAA lung perfusion (*n* = 3), ^99m^Tc MIBI Parathyroid (*n* = 3), ^99m^Tc DMSA renal (*n* = 2), ^99m^Tc -nanocolloid breast sentinel node (*n* = 1), and ^99m^Tc HMPAO-labeled leukocyte study (*n* = 1).

The study was approved by the institutional review board (IRB), and all subjects signed an informed consent form.

### Image acquisition

Patients were scanned in the analog scanner according to the clinical referral and procedure guidelines used regularly in the department. Digital SPECT studies were performed 30 to 60 min after completion of the analog study. The actual scan times used were comparable between the analog SPECT and digital SPECT.

**Fig. 2:**
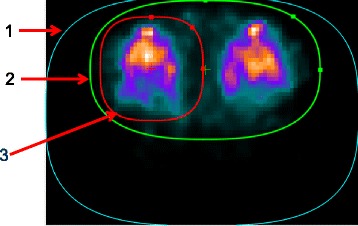
Planning a ^99m^Tc MDP SPECT study of the knees on the Valiance X12 prototype. The planning contours are overlaid on top of a transaxial slice of a low-resolution preview knee study. (1) Detector proximity contour (blue). Detectors are planned to encircle this contour. It is noteworthy that closer proximity for this patient was not possible due to the patient bed and cushions underneath the bent knees and bed straps on both sides. (2) Total activity contour (green), used to instruct the system to focus the detectors only within this ROI. (3) ROI contour (red). Used to instruct the system to dedicate more acquisition time when the detectors are focusing at this region, in accordance with user preferences (typically 50–90%)

Image acquisition workflow in the Valiance X12 prototype is as follows: The patient is positioned on an imaging table inside the scanner bore. The detectors move toward the patient until close proximity is achieved, after which the system calculates a contour containing the targeted body region. The system continues to acquire a rapid (approximately 1–2 min) low-resolution preview SPECT image to be used for the calculation of the patient-specific sampling scheme. The sampling scheme includes the order, number, duration, and angles for the detector positions, which are optimized to reduce acquisition bias resulting from insufficient statistics. To further refine the image acquisition, a target-specific ROI may be interactively placed on the preview SPECT images such that inactive areas are excluded (e.g., the imaging table). Optionally, a second ROI can also be placed within the first one, yielding a scan design in which a greater portion of imaging time is allocated to acquiring radiation emanating from the specific region in focus. Scans performed using this methodology are termed ROI-centric or ROI-focused scans. An illustration of the scan planning methodology is presented in Fig. 2.

Following the completion of the planning stage, the study includes acquisition of multiple views at each gantry position (median 180, range 100 to 500), using a dedicated mechanism for rotating the detection modules, each around its own axis (swivel motion). Once a gantry position acquisition is completed, the gantry rotates to the next position and the process repeats itself until scanning has been completed for all the planned gantry positions and full 360° angular coverage is achieved.

### Image reconstruction

Apart from brain studies, analog SPECT data was reconstructed using OSEM reconstruction [12] routinely used in the department, with 64 Gantry positions, 2 iterations, and 10 subsets, and was filtered using a Butterworth filter with cutoff frequency in the range of 0.33–0.5 and power of 10.

Analog brain studies were reconstructed using filtered back projection with Chang attenuation correction [13] and were filtered by a Metz filter with a point spread of 4 mm and power of 3.

Digital CZT SPECT data was reconstructed using the proprietary OS-MAP iterative reconstruction algorithm as previously described, with median root prior (MRP) [14].

We used 6–10 gantry positions (typically 8), 3 iterations, and 40 subsets.

For brain studies, similar assumptions to Chang-based attenuation correction, typically used for FBP reconstruction (i.e., uniform linear attenuation coefficient), were incorporated into the system model and used for attenuation correction in the iterative reconstruction.

### Interpretation of findings

All images (analog and digital) were evaluated by two experienced Nuclear Medicine physicians who were blinded to the origin of images and were graded on a scale of 1 (poor) to 4 (very good) for sharpness, contrast, uniformity, overall quality, and diagnostic confidence. When there was disagreement, the results were obtained by consensus.

Results were tested for statistical significance using the single-tailed paired *T* test.

Increased uptake of ^99m^Tc-labeled tracers (MDP, MIBI, WBC, nanocolloid) was classified as diffuse or focal, and location of the finding was noted. Reduced uptake of ^99m^Tc-labeled MAA, DMSA, and ECD was similarly described as focal or diffuse, and their location noted.

Findings from conventional SPECT and digital SPECT studies were considered congruent when both sets of images demonstrated the same findings at the same anatomical location. Discrepancies were characterized as “additional/missing” pathology (seen on Valiance X12 prototype), using the conventional SPECT study as reference.

## Results

### Overview

Fifteen analog SPECT scans were interpreted as normal, while 21 studies demonstrated abnormal findings. Thirty of the total 36 patients had congruent findings in both images (83%). There were no findings seen on conventional SPECT which were not visualized on the corresponding digital SPECT studies (i.e., no missed lesions). Two of the 15 analog studies interpreted as normal demonstrated pathology in the corresponding digital SPECT (13%). These were noted in one bone scan of the skull and one brain scan, detailed below.

### Discrepancies between analog and digital SPECT

**Fig. 3:**
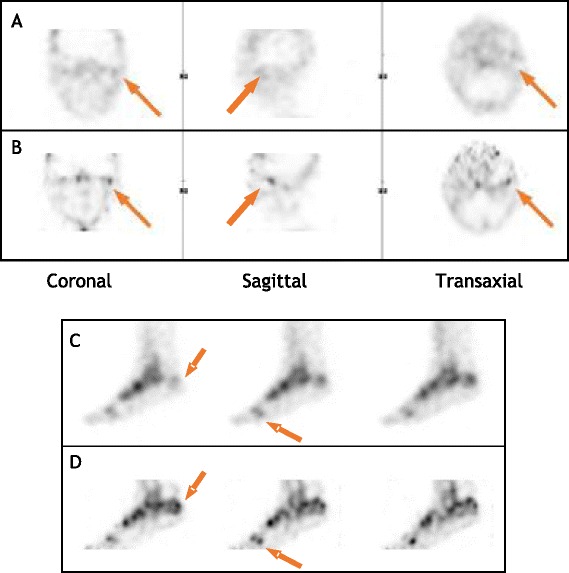
Examples of comparative clinical images (analog vs. digital CZT SPECT). Top: a 32-year-old female patient with facial asymmetry since childhood and left tempo-mandibular joint (TMJ) pain for 4.5 years referred for ^99m^Tc MDP SPECT. **a** No visible abnormality on analog SPECT. **b** Focal left TMJ uptake is seen on CZT SPECT (arrows) corresponding with clinical presentation. Bottom: A 64-year-old female patient with nonspecific foot pain referred for ^99m^Tc MDP SPECT. Representative sagittal slices of analog (**c**) and CZT (**d**) SPECT. Multiple foci, most likely osteoarthritic and stress-related (marked with arrows) are well visualized on CZT SPECT, while hardly seen or poorly visualized on analog SPECT

Overall, discrepancies were seen in 5/21 (24%) patients undergoing ^99m^Tc MDP SPECT studies. In four of these, additional foci of uptake, which were undetected on analog SPECT, were seen on the digital studies in the temporomandibular region (*n* = 1), lumbar spine (*n* = 1), and feet (*n* = 2), while in one patient, diffuse patellar uptake on analog SPECT was seen as peripheral contour uptake on the digital image. Of the 14 patients undergoing studies other than bone scans, there was only one discrepancy (7%) noted: a brain SPECT study reported to be normal per analog SPECT demonstrated asymmetric regional decrease of uptake in the left parietal lobe on digital SPECT, which was concluded to be suspicious for early onset neurodegenerative state. Examples of comparative images are presented in Fig. 3.

### Image quality comparison

**Fig. 4:**
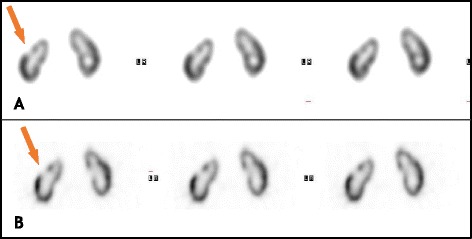
An 18-year-old male patient status post recurrent urinary tract infection referred for ^99m^Tc DMSA SPECT for evaluation of renal function. Representative sagittal slices of **a** analog and **b** digital SPECT. Right kidney cortical lesion, consistent with focal scarring (marked with an arrow), is faintly seen on analog SPECT and clearly visualized on digital SPECT

Mean sharpness was 3.79 for CZT SPECT vs. 3.26 (*p* value 0.0001) for analog SPECT, and mean contrast was 3.92 vs. 3.34 (*p* value < 0.0001) for CZT and analog SPECT, respectively. There were no statistically significant differences in uniformity (3.18 digital vs. 3.37 analog, *p* value 0.146) and in overall image quality (3.66 digital vs. 3.74 analog, *p* value 0.26). Diagnostic confidence was slightly better for conventional SPECT (3.95 vs. 3.79, *p* value 0.0416). These results are summarized in Table 1. The improved sharpness and contrast of the digital SPECT also clearly delineate a photopenic lesion as demonstrated in Fig. 4.

**Table 1:** Clinical scans grading results

		Sharpness	Contrast	Uniformity	Overall image quality	Diagnostic confidence
Analog SPECT	Mean	3.26	3.34	3.37	3.66	3.95
	Standard deviation	0.50	0.47	1.17	0.55	0.24
Digital SPECT	Mean	3.79	3.92	3.18	3.74	3.79
	Standard deviation	0.61	0.29	1.26	0.58	0.50
P value (single-tailed paired *T* test)		0.0001	< 1e-5	0.1459	0.26	0.0416

### ROI-focused digital SPECT

**Fig. 5:**
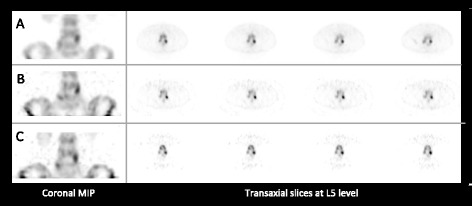
ROI-centric digital SPECT study of the lumbar spine in a 47-year-old female referred for lower back pain. A coronal maximal intensity projection (MIP) is displayed on the left side, and transaxial slices on the right side of **a** analog SPECT study, **b** digital SPECT study, and (**c**) ROI-centric digital SPECT study

Two CZT SPECT studies were performed with and without specific ROI-focusing. Visual assessment of ROI-centric images indicated improvement in both sharpness and contrast compared to the non-focused digital SPECT and further enhancement compared to the corresponding analog images (Fig. 5).

## Discussion

Gambhir et al. first described the use of a CZT-based system with unique photon collection properties and scanning geometry [1]. Combining the unique detectors with their accurate positioning and an appropriate reconstruction algorithm allowed, for the first time, the use of high sensitivity collimators, while still maintaining high spatial resolution. The increased sensitivity, together with resolution modeling in the reconstruction, allowed a better trade-off between sensitivity and resolution (i.e., improved resolution at the same noise level or lower noise at the same resolution). This improvement enabled acquisition of dynamic SPECT data (D-SPECT, Spectrum Dynamics Medical) [8]. Similar results were also reported with the GE Discovery 530c [2].

Previously performed phantom studies evaluated the reconstructed resolution and contrast of the Valiance X12 prototype CZT SPECT system, compared to an analog SPECT system fitted with high-resolution lead collimators (Discovery 670 NM/CT). Although fitted with high-sensitivity collimators, both resolution and contrast of images provided by the CZT SPECT prototype surpassed those of the analog SPECT system in optimal laboratory and in clinical-like conditions [15, 16].

The present study, a prospective single-center feasibility trial, evaluated the clinical feasibility and image quality of the CZT-based Valiance X12 prototype system in various clinical settings. The superior performance of the digital CZT SPECT system demonstrated in this clinical study was consistent with results previously reported in phantom studies [15, 16].

In agreement with the phantom results, the Valiance X12 SPECT studies consistently demonstrated improved contrast and sharpness as assessed by the readers, when compared to that of analog SPECT. All findings demonstrated in analog SPECT scans (Hawkeye and Discovery) were also detected on the digital SPECT camera. Furthermore, additional findings were seen only on the digital SPECT prototype system in three patients in this series including increased focal uptake in two bone scans (tempo-mandibular joint and foot) and decreased diffuse uptake in a brain perfusion scan, all being suggestive of underlying pathology.

There are several factors which contribute to the improved results seen in the CZT-based prototype system over the conventional analog systems, as briefly discussed in the introduction section. An improvement in the classical resolution-sensitivity trade-off is achievable due to the rich angular sampling facilitated by fine movements of the detectors. In addition, the small detector design also contributes to achieving close patient proximity, which contributes to improved image sharpness. The light weight and small footprint enable mechanical manipulation of the detectors. Another noteworthy trait of the digital SPECT is that due to the compact detector design, it is more practical to use tungsten as the collimator material. The superior photon attenuation properties of tungsten, as opposed to lead which is commonly used for analog SPECT collimators, allow a thinner septa design, which also contributes to improved system sensitivity [3]. This type of collimation also provides very good utilization of the CZT detectors by matching the collimators to the pixelated geometry. This is in contrast to scintillation cameras, with their large detectors, dictated by the analog signal acquisition pipeline. The large, heavy detectors are limited in their mechanical maneuverability which manifests in relatively coarse angular sampling and limitations in patient proximity. Under such imaging conditions, the reconstructed spatial resolution in analog SPECT systems is dominated by the choice of collimators, which are either designed for high sensitivity and low resolution, or vice versa. The LEHR collimator, very commonly used with analog SPECT and used in the present study, belongs to the latter group.

The advanced reconstruction engine, a proprietary OS-MAP reconstruction with MRP, has a critical role in image quality as well compared to the standard OSEM provided by the vendor for reconstruction of analog SPECT data. Overall image quality is determined by the acquired data, combined with the reconstruction algorithms in use. In this clinical trial, we chose to use the standard GE image reconstruction used regularly in our department and the standard reconstruction engine of the digital CZT SPECT system, comparing overall system image quality performance from a clinical perspective. Theoretically, and perhaps ideally, in order to isolate some of the variables in the comparison, the data acquired in the standard camera might have been reconstructed using the Valiance software or vice versa. However, the purpose of the present study was to evaluate the Valiance prototype as a whole and compare its overall capability to the routinely used analog SPECT units. Isolation of the impact of acquired data from that of the reconstruction engine on the overall image quality will require changes to the reconstruction software of one or both systems and may be performed in future studies.

In the present study, SPECT studies performed on the Valiance X12 prototype were found to be of better contrast and sharpness compared to same-patient studies on analog SPECT. These results were seen consistently and were apparent with abnormally increased uptake (MDP, labeled leukocytes, nanocolloid, MIBI) as well as when abnormally reduced uptake was observed (ECD, MAA, DMSA). The results described in the present study are in keeping with results reported previously for this prototype system [15, 16] and are similar to others' results evaluating a two-detector CZT-based general purpose camera [11]. Considering the performance of the dedicated cardiac CZT-based cameras, known to have improved reconstructed spatial resolution compared to conventional cameras [17], the positive results reported in general purpose CZT-based cameras such as the one evaluated here are not surprising.

The improved resolution of the dedicated cardiac cameras is well documented [18]. This, achieved together with increased count detection, is made possible by a combination of sensitive collimators with fine angular detector movements, improved patient proximity, and matching reconstruction algorithms. The prototype system described here has similar capabilities. In addition, although not tested in the present study, the motion capabilities of the novel system may also contribute to improved sensitivity compared to analog SPECT, especially for small target organs (e.g., brain), as the ability of the system to focus the detectors on the imaged target allows refraining from practically inactive detector surface.

As described, one patient volunteered to undergo both standard digital and ROI-focused imaging, indicating the further improvements that specific focusing can provide. More data is required to properly assess the clinical benefits of ROI-focused scanning, a unique capability of this CZT-based digital SPECT system.

Significant increase of sensitivity in CZT-based dedicated cardiac cameras compared to conventional analog systems allows for a reduction in scan times and/or administered dose [10, 19–21]. The results of the present study indicate that Valiance X12 prototype can provide images of diagnostic quality with improved contrast and sharpness compared to conventional analog technology. Further investigations are warranted to assess the sensitivity of this system and to demonstrate that similar reduction in scanning times or doses is also feasible.

Despite the better overall grades given to digital SPECT images compared to analog SPECT, the diagnostic confidence averaged slightly higher for conventional SPECT images. Readers are naturally confident when reading scans with the characteristic appearance they are accustomed to in their daily practice, and the sharper digital images may require additional training and education to increase reader confidence. As common with many technological advances, an adjustment period may be required before transitioning to full-time use of digital SPECT for clinical practice.

A limitation of the present study was the design which restricted the order of imaging, with all patients being scanned in the established analog camera first. This approach was adopted to address requirements by the ethic committee, given the fact that the digital SPECT is at a prototype level, and in order to make sure the established analog SPECT procedure is performed following the standard protocol. Furthermore, the different-sized fields of view of the two systems hampered effective blinding of the reviewers to the origin of the images. Further studies might be randomized with some patients being imaged in the CZT system first, to allow for any effect of pharmaceutical kinetics between the scanning procedures. The additional lesions seen on the digital scans are believed to be true findings, and not artifacts, perhaps afforded by the improved contrast and sharpness of CZT SPECT images, as they correspond to the clinical indications. However, there is no correlative evidence which could conclusively determine the nature of these findings. This is another limitation of the present study that will need to be addressed in future work. As discussed above, the clinical images in this study are the result of the camera acquisition, combined with the reconstruction engine in use. Additional studies may be performed in order to isolate these two factors in each of the cameras and better understand their separate contribution to overall image quality.

Finally, images were evaluated visually by two observers, which may have created a bias. Further studies may entail multiple observers and/or evaluation methods not biased by the human eye.

## Conclusions

Despite using high-sensitivity collimation, SPECT images in selected clinical studies obtained from the Valiance X12 prototype were of higher resolution and contrast compared to analog SPECT. General purpose digital SPECT systems with high-sensitivity collimators, with improved image quality compared to analog SPECT technology, may have a significant clinical impact. Further studies are needed to evaluate the diagnostic performance of the system in large patient cohorts and in various clinical settings. Such studies will evaluate how the system’s unique properties contribute to its superior performance and will determine the clinical impact of this new technology.

## Abbreviations

99mTc: Technetium
CZT: Cadmium zinc telluride
DMSA: Dimercaptosuccinate
ECD: Ethylene cysteine dimer
MDP: Methylene di-phosphonate
ROI: Region of interest
SPECT: Single-photon emission computed tomography
